# A Ligation of the Lacrimal Excretory Duct in Mouse Induces Lacrimal Gland Inflammation with Proliferative Cells

**DOI:** 10.1155/2017/4923426

**Published:** 2017-08-10

**Authors:** Ying Liu, Masatoshi Hirayama, Tetsuya Kawakita, Kazuo Tsubota

**Affiliations:** ^1^Department of Ophthalmology, Keio University School of Medicine, Shinjuku, Japan; ^2^Regulatory Biology Laboratory, Salk Institute for Biological Studies, La Jolla, CA, USA; ^3^Department of Ophthalmology, Kitasato Institute Hospital, Kitasato University, Minato, Japan

## Abstract

The lacrimal gland secretes tear fluids to ocular surface, which plays an indispensable role in maintaining the health of the ocular epithelia and protecting the ocular surface from the external environment. The dysfunction of the lacrimal glands causes dry eye disease due to a reduction in tear volume. The dry eye disease is becoming a popular public disease, for the number of patients is increasing, who have subjective symptom and loss of vision, which affect the quality of life. Inflammatory change in the damaged lacrimal gland has been reported; however, a major challenge is to establish a simple animal model to observe the changes. Here, we demonstrated an injury model by ligating the main excretory duct of the lacrimal gland, which is a simple and stable way to clearly understand the mechanism of lacrimal gland inflammation. We observed the process of injury and proliferation of the lacrimal gland and detected a population of lacrimal gland epithelial cells with proliferation potential which were also nestin-positive cells following duct ligation. This study successfully established an injury model to observe the tissue injury process of the lacrimal gland, and this model will be useful for analysis of the inflammation and proliferation mechanism in the future.

## 1. Introduction

The lacrimal gland (LG) is a secretory gland that secretes tear fluids to maintain the health of the ocular surface epithelium and protect the ocular surface from the external environment [1]. Dysfunction of the LG leads to dry eye disease (DED), an ocular surface disease characterized by insufficient tear production and damaged epithelium [2]. A significance to treat DED based on the understanding on mechanism on pathological changes in the LG has increased because DED has become one of common eye diseases in elderly people that causes subjective symptoms including discomfort as well as objective signs such as foreign body sensation, ocular irritation, and blurred vision, which affect the quality of life [3–5].

The LG is composed of various kinds of cells such as acinar, duct, and myoepithelial cells. The acini express water channel proteins including aquaporin 5 (AQP5) for aqueous tear secretion [6]. The myoepithelial cells that surround the acini help to secrete tear contents from acini in response to nerve stimulation [6]. To analyze the mechanism of tear decreasing after injury to the LG, several animal models, which could induce inflammation of the LG, have been proposed [7]. The single injection of IL-1, a cytokine, to the LG induced the inflammation partially and temporarily, and it caused reversible change after 7 days [8]. The reports indicated a regeneration capacity in the partial damaged LG [9]. An establishment of animal models for an efficient LG injury has been required to elucidate pathological changes against persistent injuries to the LG [10–12].

Duct ligation of organs has been a conventional and promising method to induce organ dysfunctions including inflammation and fibrosis by obstructing the fluid pathway, blood vessels in the bile duct, and liver arteries [13, 14]. Obstruction of the fluid pathway itself has recently been recognized as a novel pathology to lead meibomian gland dysfunctions [15–17]. However, it has been still controversial that the duct ligation of the lacrimal gland excretory duct can cause lacrimal gland inflammation efficiently in an animal model.

Here, we demonstrated a mouse model to induce LG injury by the ligation of the lacrimal gland excretory duct. The duct ligation caused stable and throughout injury of the LG; we observed the process of injury and proliferation of the LG and detected a population of LG epithelial cells with proliferation potential, which was also a population of cells positive for nestin, the stem/progenitor cell marker. Our current study provides evidence of a LG injury pattern by duct ligation, and this model will be useful for analysis of the mechanism and process of lacrimal gland inflammation and identification of a candidate stem/progenitor cell of the LG in the future.

## 2. Methods

### 2.1. Animals

Thirty-nine male C57BL/6Jc1 WT mice (7-8 weeks old) were purchased from CLEA Japan Inc. Mice were feed in a controlled environment following the guidelines of Keio University. The care and handling of the animals were performed in accordance with the NIH guidelines. All of the experimental protocols were approved by the Animal Care and Use Committee of the Keio University (Approval number 09167).

### 2.2. Duct Ligation of the Main Lacrimal Gland Excretory Duct

**Figure 1:**
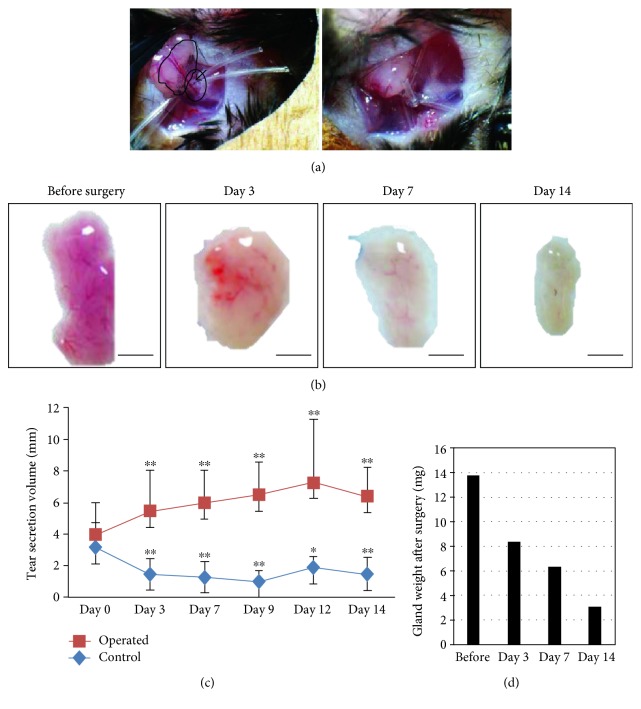
The lacrimal duct ligation in mouse. (a) The photos of the lacrimal duct ligation procedure in mice. The white circle showed the excretory duct of the LG and the black circle showed the main LG. The procedure before ligation (left) and after ligation (right) was shown. (b) The macroscopic changes in the LG before and after ligation. The scale bar is 2 mm. (c) The change in the average of tear secretion volume before and after ligation. Each group has more than 20 samples. ^∗^*p* < 0.05, ^∗∗^*p* < 0.01. (d) The change in the gland weight before and after surgery. Each group has more than 20 samples.

We established a mouse injury model by ligating the main excretory duct of the LG; this procedure induced injury to the tissue of the LG, which is shown in Figure 1(b). Animals were anesthetized using 2 mg/mL ketamine (Lot: V29D; Ciba Specialty Chemicals Holding Inc.) and 2 mg/mL xylazine (Lot: KP035HU, Bayer Holding Ltd.) mixed in saline (10 *μ*L/g) by intraperitoneal injection. A small incision (less than 8 mm) was made to the right side face skin. We gently lifted up the LG to expose the main excretory duct and then ligated the duct together with the vessel using a silicon tube (0.2 × 0.3 mm, 1-8194-02 No.2, Justis, Japan). After the operation, the skin underwent two interrupted sutures with 10-0 nylon (Lot: 03-9315, Alcon). The left LG was not operated on and served as a control. Mice in the sham group had the right side skin cut but did not undergo LG ligation. The animals were sacrificed 3, 7, or 14 days after ligation. LGs were removed immediately after sacrifice.

### 2.3. Measurement of Tear Secretion

A phenol red-impregnated cotton thread was used to measure the aqueous tear production of experimental animals without anesthesia. The cotton thread was placed on the inner canthus gently without touching the ocular surface for 30 s, and the length of the part which was stained red was measured as previously reported [18]. We measured aqueous tear production before operation and 3, 7, 9, 12, and 14 days after surgery.

### 2.4. Sample Collection and Weight Measurement

All LG specimens were removed from the surrounding tissue entirely, washed in PBS, dried, and weighed immediately with an electronic balance. The LG specimens were then cut in half to be used for real-time PCR, and the other half was fixed in OCT, immediately frozen in liquid nitrogen, and stored at −80°C until stained.

### 2.5. Histological Analysis of the LG

LG specimens were cut into sections (5-6 *μ*m) for H&E and immunohistochemical staining. For H&E staining, the tissue sections were fixed with 10% formalin for 10 min and then stained with hematoxylin and 1% eosin. For immunohistochemical staining, the fresh tissue sections (5 *μ*m) were fixed with 4% paraformaldehyde (PFA; Wako, Osaka, Japan) in PBS and then blocked with 10% normal donkey/goat serum (IHR-8135, ImmunoBioScience Corp., USA; S-1000, Lot: Y0322, Vector Laboratories, CA, USA) with 0.01% Triton X-100 (35501-15, Nacalai) in PBS. After blocking, the tissue sections were incubated with the appropriate primary antibody overnight. The next day, after washing with PBS, the sections were incubated with the secondary antibody (1/300) and 0.1% Hoechst (1/1000) for 1 hour and observed. The following are the primary antibodies used for immunostaining: rabbit cytokeratin14 (K14, 1/1000; PRB-155p, Covance); mouse *α*-smooth muscle actin (*α*-SMA, 1/500, ab18640, Abcam), which is a marker of myoepithelial cells; rabbit AQP5 (1/100, SC-28628, Santa Cruz); and nestin (1/80; AF2736, R&D), which is a neural stem cell marker; to evaluate the proliferation of acinar and duct cells after ligation, LG specimens were incubated with PCNA (1/100; M0879, Dako) as a cell proliferation marker. Anti-PCNA antibody was performed by a DAB staining kit (Lot: 415172, Nichirei Biosciences Inc.). A TUNEL assay was performed using the ApopTag Red apoptosis detection kit (Millipore, Bedford, MA, USA), according to the manufacturer's protocol. Histological sections were observed under the fluorescence microscope (BIOREVO BZ-9000) and Imager (Axiocam; Carl Zeiss Inc.). Imaging of the whole dish was captured and processed using BIOREVO (Keyence Corporation, Osaka, Japan), and the nestin-positive and CD45-positive area was calculated as pixel numbers.

### 2.6. RNA Extraction and Real-Time PCR

Total RNA was extracted from cells using TRIzol reagent (Invitrogen, Carlsbad, CA, USA) following the manufacturer's protocol. A NanoDrop 1000 Spectrophotometer (Thermo Scientific) was used to measure RNA concentration. Synthesis of cDNA from total RNA was performed using ReverTra Ace® qPCR RT Master Mix (FSQ-201, Toyobo, Japan). Quantitative real-time PCR was performed using the StepOnePlus equipment (Applied Biosystems) with TaqMan® Fast Universal PCR Master Mix (2X), without AmpErase® UNG (Applied Biosystems), and predesigned primers for cytokeratin 14, *α*-SMA, AQP5, nestin, and glyceraldehyde-3-phosphate dehydrogenase (GAPDH). The ΔΔCT value calculated by StepOne Software (v2.2.2) was used to evaluate the mRNA levels and was normalized to GAPDH mRNA.

## 3. Results

### 3.1. Macroscopic Changes in the Injured LG by the Duct Ligation

We first demonstrated the ligation of the mouse lacrimal excretory duct to investigate whether the procedure could cause LG injury (Figure 1(a)). After the ligation procedure, we analyzed the macroscopic changes in the LG before surgery and at days 3, 7, and 14 after surgery. The macroscopic observation showed that at 3, 7, and 14 days postligation, the LG was diminished and hardened and the transparency decreased (Figure 1(b)). To confirm the ligation effect of the duct, we also analyzed the change in tear secretion volume before and after surgery. We performed the ligation procedure on the right eyes, and we used the left eye as the control group without duct ligation (sham surgery group). In the right eye with duct ligation, tear secretion at 3, 7, 9, 12, and 14 days after surgery was significantly decreased compared with that in the eye in the sham surgery group (Figure 1(c)). Interestingly, the control showed a significant increase compared with day 0 at each time point due to compensation. We next analyzed the weight change during the procedure. The weight of the LG was decreased after surgery during the procedure (Figure 1(d)). These results indicated that the ligation of the lacrimal excretory duct was a success and could induce throughout injury in the LG.

### 3.2. Histological Changes in the LG after the Duct Ligation

**Figure 2:**
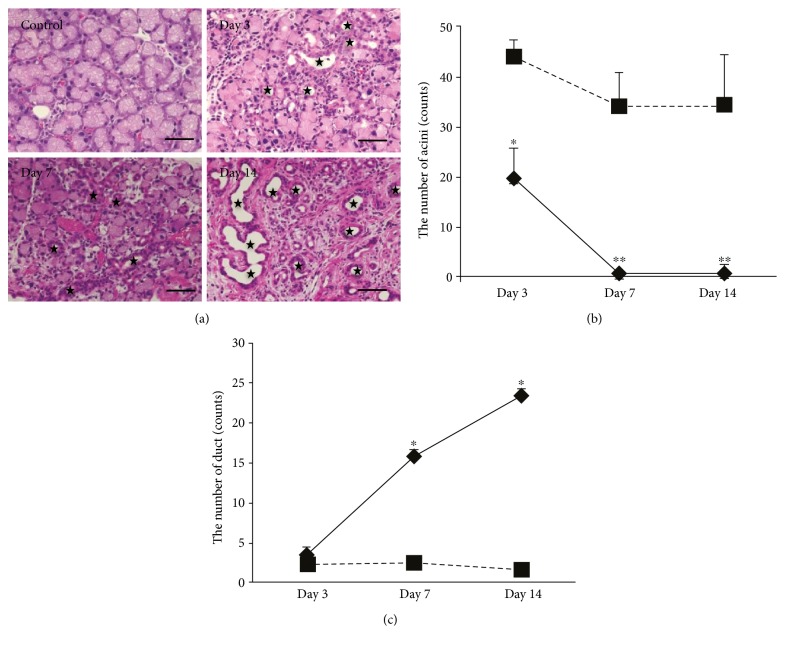
Histological changes in the LG after the ligation. (a) The representative images of the H&E staining of the LG before and after surgery. ★ showed the enlarged and generated duct cavities. The scale bar is 50 *μ*m. (b) The change in the average number of acini after surgery. ^∗^*p* < 0.05, ^∗∗^*p* < 0.01. (c) The change in the average number of duct after surgery. ^∗^*p* < 0.05.

We next investigated the pathological changes in the duct-ligated LG by histological analysis. We observed histological changes in the LG before surgery and following 3, 7, and 14 days after ligation by H&E staining. Before surgery, the H&E staining showed the normal secretory gland histology including acini and ducts. After surgery, the results showed the infiltration of inflammatory cells into the connective tissue and around the duct area, especially at day 3 postligation. Extensive injury was observed at day 3, and the peak of the injury was observed at day 7. After 14-day surgery, almost all the acini were diminished, with only few remaining; on the other hand, many residual and newly formed ducts were observed (Figure 2(a)). Quantitative analysis of acini and duct that unite following ligation also showed that the number of acini was significantly decreased and the number of ducts was significantly increased (Figures 2(b) and 2(c)). These results indicated that the LG after the duct ligation induced inflammation and atrophic changes, whereas some proliferative changes such as a duct increase occurred.

### 3.3. mRNA Expression Changes in the LG after the Duct Ligation

**Figure 3:**
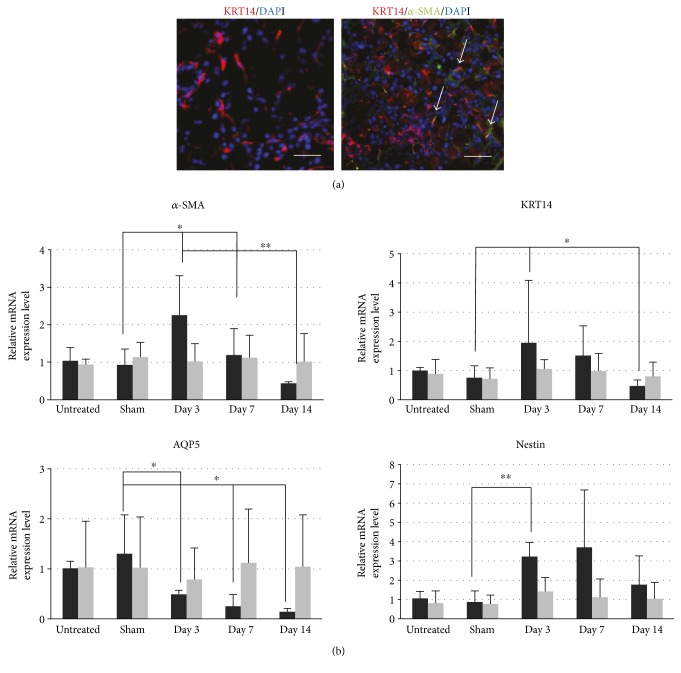
Expression changes in representative markers in the LG after surgery. (a) The histoimmunochemical staining of KRT14 and *α*-SMA in the normal LG (red showed the KRT14, green showed the *α*-SMA, and arrow showed the cells with both expression). The scale bar is 50 *μ*m. (b) The difference of relative mRNA expression level of the representative markers before and after surgery. ^∗^*p* < 0.05 and ^∗∗^*p* < 0.01.

To clarify the proliferative changes in inflammatory status in the LG after the duct ligation, we performed real-time PCR analysis to observe gene expression changes in the LG by a time course. We analyzed the relative mRNA expression changes about cytokeratin 14 (KRT14) that was expressed in duct and myoepithelial cells, aquaporin 5 (AQP5) that was expressed in acinar lumens, and *α*-SMA that was expressed in myoepithelial cells as representative markers in duct, acinar, and myoepithelial cells (Figure 3(a)). The expression of KRT14 and *α*-SMA in the LG with duct ligation significantly increased temporarily with a peak at day 3. On the other hand, the expression of AQP5 was significantly decreased after the surgery. We next investigated the expression of nestin, a stem/progenitor marker, in the LG after duct ligation. The relative expression level of nestin also increased with a peak at day 7 (Figure 3(b)). These results indicated that the LG after the duct ligation induced not only atrophic inflammation but also proliferation of cells. The cells expressing KRT14 and *α*-SMA proliferated immediately after injury, while cells expressing nestin increased following that.

### 3.4. Immunohistological Analysis on Inflammation and Proliferative Changes in the Duct-Ligated LG

**Figure 4:**
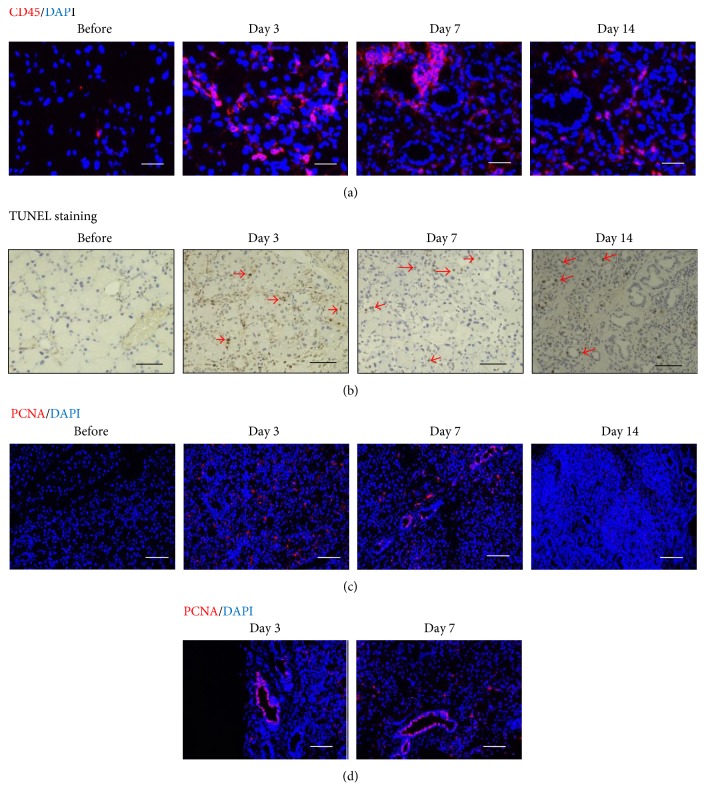
The inflammation and proliferation of the LG after surgery. (a) The time-course observation of cells with CD45 expression before and after surgery (red showed the CD45). The scale bar is 50 *μ*m. (b) The time-course observation of cell apoptosis by TUNEL staining before and after surgery (red arrows showed the stained cells). The scale bar is 50 *μ*m. (c) The time-course observation of cells with PCNA expression before and after surgery (red showed the PCNA). The scale bar is 50 *μ*m. (d) The enlarged image of PCNA-positive cells at days 3 and 7 after surgery (red showed the PCNA). The scale bar is 20 *μ*m.

To clarify the detail of the effect of duct ligation on the LG, we have analyzed inflammation and proliferative status in the LG after the procedure by immunohistological analysis. First, we examined the inflammation condition at each time point using CD45, a lymphocyte common antigen, which is expressed on all leukocytes. We observed an infiltration of large number of CD45^+^ cells at day 3 and day 7, and it gradually diminished at day 14 following the ligation (Figure 4(a)). We next evaluated cell apoptosis induced by ligation by TUNEL staining. A few TUNEL-positive cells were found in acini and ducts at day 3, and TUNEL-positive cells increased in the luminal layer of the ducts at day 7, converging in one area, which was near the ligation site, and duct cell in the basal layer survived. TUNEL was almost negative at day 14 after surgery (Figure 4(b)). These suggested that the peak of inflammation occurred in the early stage and the inflammation decreased gradually.

To evaluate the proliferative process in the LG after the duct ligation, we analyzed the expression of PCNA, which was expressed in proliferative cells. The PCNA-positive (PCNA^+^) cells were found in the basal layer of the stratified duct epithelium above the basement membrane at day 3 and also in interstitial tissues. At day 7, most PCNA^+^ cells were found in acini and only a few were in the basal layer of the ducts. At day 14, the PCNA^+^ cells were localized in small simple duct epithelium (Figures 4(c) and 4(d)). These results suggested that proliferative cells in the injured LG may contribute to the formation of the small ducts after the inflammation.

### 3.5. Expression of Nestin in the Injured LG after the Duct Ligation

**Figure 5:**
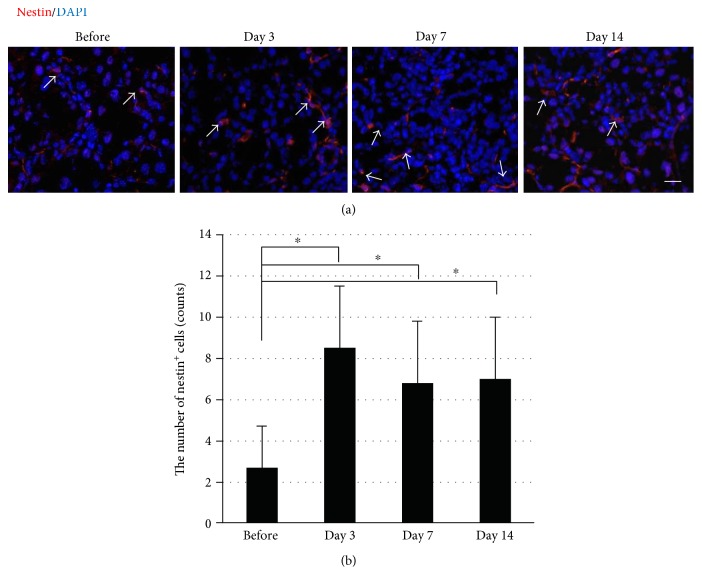
The increase in nestin-positive cells in the LG after surgery. (a) The time-course observation of cells with nestin expression before and after surgery (red and arrows showed the nestin). The scale bar is 50 *μ*m. (b) The change in the average number of cells per visual field with nestin expression before and after surgery. More than 10 samples in each group were included. ^∗^*p* < 0.05.

Nestin, a stem cell marker of the central nervous system, has been used as a stem/progenitor marker of many glandular tissues including those of the mammary gland, pancreas, hair follicle, salivary gland, and sweat gland [19–24]. Therefore, we further investigated whether cells with the expression of nestin were found in the proliferative status in the LG after the duct ligation. Immunostaining revealed that nestin-positive (nestin^+^) cells increased in the interstitial tissues in the LG with an elongated morphology at days 3, 7, and 14 after surgery, whereas it was a few in the normal LG (Figure 5(a)). The number of nestin-positive cells was significantly increased at days 3, 7, and 14 after ligation compared with that of the control (Figure 5(b)). To analyze the origin of them, we performed the immunostaining with KRT14 that was expressed in acinar and myoepithelial cells and *α*-SMA that was expressed in myoepithelial cells in the LG, and they were not expressed in nestin^+^ cells (data not shown). These results suggested that the proliferation process after inflammation in the LG was supported by proliferative cells such as PCNA^+^ cells in the basal layer in duct and nestin^+^ cells in interstitial tissues in the LG.

## 4. Discussion

In this study, we have demonstrated the ligation of the LG excretory duct to establish a tissue injury model of the adult mouse LG. Our model could successfully induce the atrophic changes throughout the LG after the surgery. The procedure easily created injury in the LG over a time course and showed the inflammation and proliferative process after surgery, which is almost consistent with the other LG injury model. Our study clarified the time course change in the lacrimal gland after the duct ligation and showed the induced inflammation at the early stage and proliferative changes with PCNA^+^ cells and nestin^+^ cells. This model will be useful for investigation on character in such proliferative cells expressing a stem cell marker in the LG.

Animal models for efficient tissue damage have been reported for investigation on mechanism of inflammation, regenerative potential of organs, and isolation of stem/progenitor cells from the target organs [8, 25–29]. Previous studies have shown that a partial radiation to salivary glands in mice leads to proliferation of cells, which have an ability to form salispheres for cell injection therapy to restore the salivary gland functions [30]. In the LG, some animal models for tissue damage using cytokines and bone marrow transplantation have been established, and they have revealed mechanisms of induced inflammation and regeneration abilities in the LG after injury [7, 31, 32]. The duct ligation procedure is simple and stable to lead to tissue inflammation in the liver, bile ducts, and salivary glands [13, 33–35]. However, the details on the effect of the duct ligation on the mouse LG have been unclear because no studies about them have been reported. We have successfully demonstrated a new tissue injury model of the mouse LG by duct ligation in this study.

Mechanisms of tissue damage in this model were based on both tear flow obstruction and ischemic changes by the ligation of duct and an artery running along with the duct [34]. We could confirm them by a tear flow decrease after the ligation and white color change in the LG in macroscopic observation. Anatomically, the major artery for the LG flows from two directions, front (along with the duct) and back sides [36]. The artery along with the duct supplies approximately 60% of blood flow to the LG. In this model, because the rest of blood supply was maintained [36], the LG could not lead to necrosis immediately. Such a partial injury status is crucial to observe inflammation status and proliferation status after the injury [8]. Our model could lead to proliferative status after inflammation as consistent with the previous partial injury model in the LG.

Current studies on regenerative medicine have revealed that proliferating cells after injury have a possibility to reconstruct the damaged tissues [37]. In our study, the small duct structure increased after surgery, which indicated a part of regeneration capacity of the LG. In the PCNA labeling experiment, we found that the PCNA^+^ cells were first detected in the basal layer of the ducts at day 3, the PCNA^+^ cells proliferated at day 7, and then most PCNA^+^ cells were found in the simple layer of the ducts at day 14. The temporal increase in PCNA^+^ cells at the basal layer of the duct suggested the contribution to the increase in the small ducts. Nestin, the intermediate filament protein, has been focused as a stem/progenitor marker of the central nervous system and the hair follicle [24]. Studies using nestin-driven GFP (ND-GFP) transgenic mice have revealed the possibility of the nestin^+^ hair follicle stem cells as an autologous source of adult stem cells to repair damaged tissues including those of nerves and cardiac muscles [38–40]. In previous reports, nestin^+^ cells, which were located in interstitial tissues, play an essential role in the repair of the LG after injury. Nestin^+^ cells have been recognized as multilineage progenitor cells and gland-derived stem cells [22, 23]. In our model, the nestin^+^ cells were also located in the interstitial tissues between acini and ducts and they increased after the duct ligation successfully. Interesting finding would be provided by the analysis of pluripotency and regeneration capacity of the nestin^+^ cells in the LG.

## 5. Conclusion

In summary, we demonstrated the ligation of the LG excretory duct to induce partial tissue injury in the mouse LG. The current model could successfully induce the inflammation and proliferation process with the increase in cells expressing a stem cell marker. This mouse model will be a useful tool to observe the inflammation process and analyze the origin and regeneration capacity of proliferative cells, such as nestin^+^ cells, in the LG to reveal mechanism to restore tissue damages.
